# Protective multi-stressor interactions in the Anthropocene: Key considerations for investigating cross-tolerance in a conservation context

**DOI:** 10.1093/conphys/coaf052

**Published:** 2025-07-30

**Authors:** Essie M Rodgers, Simone Baldanzi, Michael Collins, W Wesley Dowd, Lauric Feugere, Giovanna Mottola, Fanny Vermandele, Daniel F Gomez Isaza

**Affiliations:** School of Environmental and Conservation Sciences, College of Environmental and Life Sciences, Murdoch University, 90 South Street, Murdoch, WA 6150, Australia; Centre for Sustainable Aquatic Ecosystems, Harry Butler Institute, Murdoch University, 90 South Street, Murdoch, WA 6150, Australia; Laboratorio de Ecofisiología y Ecología evolutiva marinas (e°CO2lab) and Centro de Observación y Análisis del Ambiente Costero (COSTA-R), Universidad de Valparaíso, Avenida Gran Bretaña, 1111, cerro Playa ancha, 2340000, Valparaíso, Chile; Estación Costera de Investigaciones Marinas and Millennium Nucleus for the Ecology and Conservation of Temperate Mesophotic Reef Ecosystems (NUTME), Pontificia Universidad Católica de Chile, Osvaldo Marín 1672, Las Cruces, El tabo, 2690000, Chile; Marine Biology and Ecology Research Centre, School of Biological and Marine Sciences, University of Plymouth, Drake Circus, Plymouth PL4 8AA, UK; School of Biological Sciences, Washington State University, 100 Dairy Road, Pullman, WA 99164, USA; Laboratory of Marine Ecological and Evolutionary Physiology, Department of Biology, Chemistry and Geography, University of Quebec in Rimouski, 300 Allée des Ursulines, Rimouski, QC G5L 3A1, Canada; Department of Environmental and Biological Sciences, University of Eastern Finland, Yliopistonkatu 7, P.O. Box 111, Joensuu FIN-80101, Finland; Laboratory of Marine Ecological and Evolutionary Physiology, Department of Biology, Chemistry and Geography, University of Quebec in Rimouski, 300 Allée des Ursulines, Rimouski, QC G5L 3A1, Canada; Harry Butler Institute, Murdoch University, 90 South St, Murdoch, WA 6150, Australia; Australian Institute of Marine Science, Indian Ocean Marine Research Centre, University of Western Australia, Crawley, WA 6009, Australia

**Keywords:** Abiotic stressor, conservation management, cross-protection, cross-talk, environmental variability, inducible stress tolerance, multiple stressors, preconditioning, species resilience, stressor interactions

## Abstract

In the Anthropocene, species are increasingly faced with multiple stressors that are more severe and less predictable than before. While multiple stressors often interact to affect organisms negatively, sometimes these interactions can be beneficial, enhancing resilience through cross-protection. Cross-protection interactions occur when exposure to one stressor, such as elevated temperature, enhances an organism’s tolerance to a different stressor, like hypoxia, through shared protective mechanisms or signaling pathways. Understanding the potential for cross-protection to combat rapid and diverse environmental change is crucial for conservation, as it potentially alters the predicted consequences of such change. Here, we outline 10 key considerations for investigating cross-protection in a conservation context. These considerations include the importance of stressor intensity and timing, recognizing species-specific and sex-specific responses, and embracing temporal variability in environmental stressors. Additionally, predictions will depend upon uncovering the underlying mechanisms of cross-protection by integrating emerging approaches like omics and meta-analyses. By better understanding—and in some cases explicitly leveraging—cross-protective interactions, conservation practitioners may be able to develop more effective management plans to enhance species resilience, potentially mitigating the immediate effects of emerging stressors. These insights are vital for guiding future research directions and informing conservation policies and management practices to preserve biodiversity in the Anthropocene.

## Introduction

Species are being forced to contend with a growing list of stressors as human pressures on ecosystems escalate. Here we define stressors as any change in a species’ environment that negatively impacts fitness or performance (Schulte, 2014). The growing burden of multiple stressors means that we must consider how stressors interact to affect imperiled species and ecosystems—a situation that poses a pressing conservation challenge. With this goal in mind, a flurry of multi-stressor research has been generated over the last two decades (Todgham and Stillman, 2013; Gunderson *et al*., 2016; Orr *et al*., 2024; Rodgers and Gomez Isaza, 2024; Gutierrez *et al*., 2025). Stressors can interact in a variety of ways, but the incidence of negative interactions (i.e. negative synergisms) between concurrent stressors are overreported in ecological literature and often claimed without explicit testing (Côté *et al*., 2016). This focus on synergisms has contributed to the misconception that all stressor interactions are deleterious. We are finding, though, that not all stress is bad, and exposure to one mild stressor can sometimes ‘prepare’ species for future, distinct stressors.

**Figure 1:**
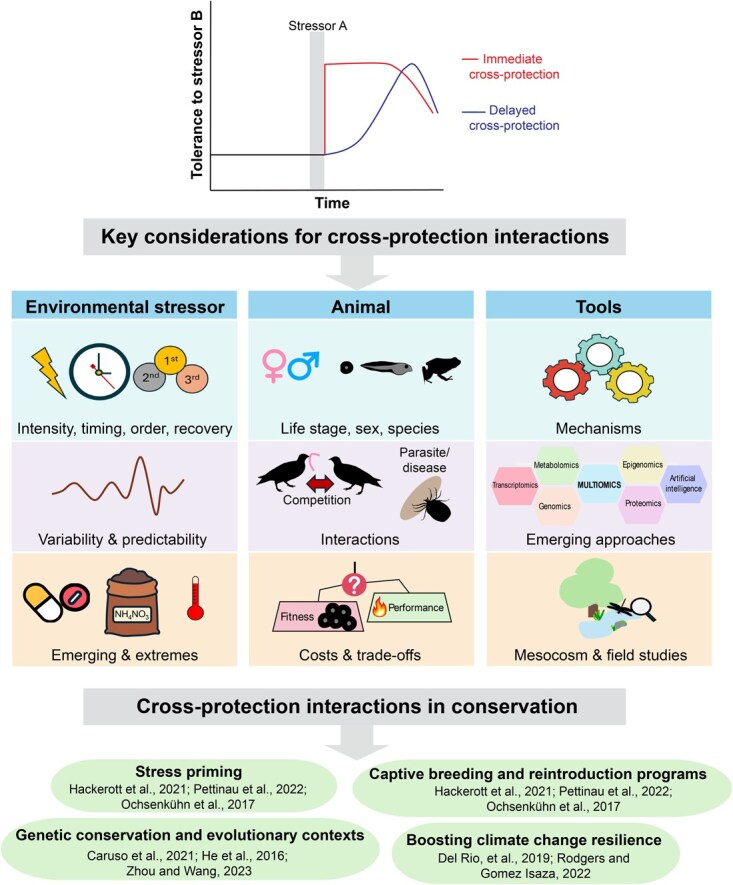
**Conceptual framework of cross-protection stressor interactions, key investigative considerations and conservation applications.** Cross-protection occurs when exposure to one stressor (Stressor A) enhances tolerance to another stressor (Stressor B). This response may be immediate or require a recovery period for protective mechanisms to become active. Environmental factors—such as stressor type, intensity, timing and predictability—can influence these interactions, which have been observed between naturally co-occurring stressors as well as emerging stressors like microplastics. Cross-protection varies across species, life stages and sexes, sometimes involving fitness trade-offs. Cutting-edge tools such as multiomics and artificial intelligence (AI) hold promise for advancing our understanding of these dynamics. Ultimately, insights into cross-protection can inform conservation strategies, including stress priming and targeted captive breeding programs.

Encountering one stressor can sometimes prime (‘prepare’) an organism for future stress of a different nature, a phenomenon termed cross-protection (Rodgers and Gomez Isaza, 2021; Fig. 1). Cross-protection arises when stressors activate shared protective mechanisms (termed ‘cross-tolerance’) or signaling pathways (termed ‘cross-talk’) (Sinclair *et al*., 2013). For example, exposure to heat stress can often increase hypoxia resistance in vertebrates, because these stressors can be countered through shared mechanisms (e.g. hypoxia-inducible factor-1 [HIF-1] protein levels, increases in blood oxygen carrying capacity, and remodeling of the cardiorespiratory system) (Ely *et al*., 2014). Exposing rats (*Rattus norvegicus*) to heat stress for 1 month, for example, significantly increased hypoxia tolerance due to greater erythropoietin expression and higher HIF-1 protein levels (Maloyan *et al*., 2005). Similarly, channel catfish (*Ictalurus punctatus*) exposed to hypoxia for 1 week showed improved heat tolerance compared to control fish, and this improvement was linked to remodeling of the cardiovascular system (Burleson and Silva, 2011). Cross-protection (encompassing both cross-talk and cross-tolerance) has been documented in a wide diversity of taxa spanning the bacteria, fungi, plant and animal kingdoms (Rodgers and Gomez Isaza, 2021). These protective interactions have also been observed in a range of habitats including intertidal, freshwater, rainforests and polar zones, and in response to a variety of both abiotic and biotic stressors (Rodgers and Gomez Isaza, 2021).

It has been hypothesized that cross-protection interactions may have evolved in response to predictable stressor cycles in habitats, leading to the prediction that they should be most frequently observed between stressors that predictably co-vary in nature. For instance, many polar terrestrial species face dry conditions paired with subzero temperatures, and cross-protection between desiccation and cold stress is widespread in polar insects (Sinclair *et al*., 2013). The cross-protection is achieved, at least in part, via overlapping mechanistic responses (e.g. osmoprotectants, cyroprotectants and molecular chaperones; Sinclair *et al*., 2013) Likewise, cross-protection has been documented in some tidepool/splashpool species in response to stressors that fluctuate predictably with changing tides, like temperature and salinity spikes, and hypoxia paired with desiccation (Todgham *et al*., 2005; Denny and Dowd, 2022; von Weissenberg *et al*., 2022).

Remarkably, cross-protection interactions have been observed not only in response to natural environmental stressors, but also among novel, emerging stressors such as pharmaceuticals, pesticides and climatic extremes. For example, heat tolerance in *Daphnia magna* increased following two generations of exposure to polystyrene microplastics (Chang *et al*., 2023). Such findings suggest that species may develop enhanced stress resilience as a byproduct of dealing with other, unrelated environmental challenges. Understanding interactions among emerging stressors is becoming increasingly pressing, particularly for conservation practitioners. Cross-protection may be a highly effective mechanism buffering populations from the immediate effects of novel stressors, and it may allow time for genetic adaptation to take hold. Thus, cross-protective mechanisms may not only enhance an organism’s survival prospects but also influence broader ecological dynamics and evolutionary processes.

Research on cross-protection interactions is growing rapidly, but this topic is laden with complexity. Protective interactions are dependent on the intensity, timing and order of stressor interactions and can vary among species, life stages and sexes. While much of the research has focused on abiotic stressors, biotic stressors remain underexplored and require further investigation. Understanding the underlying mechanisms of cross-protection is challenging, yet crucial, and emerging methods such as omics, meta-analyses and modeling hold promise in uncovering these mechanisms. Moreover, the trade-offs associated with cross-protective phenotypes need more attention, as these could have significant ecological and evolutionary implications. Finally, while laboratory studies provide valuable insights, cross-protection needs to be investigated in natural field settings to understand how these interactions play out in real-world environments. This perspective will discuss 10 key considerations (Fig. 1) for practitioners wishing to incorporate the potential for cross-protection into conservation, emphasizing the importance of embracing the complexity of cross-protection interactions and investigating these stressor dynamics from multiple angles to gain a deeper understanding of their role in conservation in rapidly changing environments.

## Stressor Intensity, Timing, Order and Recovery Matter

The intensity, timing and order of stress exposure are critical factors influencing cross-protection mechanisms. These elements often determine whether stress exposure will result in beneficial protective interactions or deleterious interactions. Mild stress exposure typically induces protective mechanisms, like the upregulation of heat shock proteins, without overwhelming the organism, thereby boosting resilience to subsequent stress. For example, low sublethal concentrations of certain chemical contaminants induced cross-tolerance to insecticides in larval wood frogs, whereas higher, sublethal concentrations led to negative outcomes (*Rana sylvatica*; Jones *et al*., 2024). Similarly, exposure to mild salinity stress (10 ppt) increased heat tolerance in juvenile Chinook salmon (*Oncorhynchus tshawytscha*), but severe salinity stress (33 ppt) provided no benefit (Rodgers and Gomez Isaza, 2022). Stress exposure can often be either too mild to trigger protective mechanisms or too severe, following the ‘Goldilocks principle’ where stressor intensity needs to be ‘just right’. For instance, research on tidepool sculpins (*Oligocottus maculosus*) showed that a +10°C heat shock did not affect salinity tolerance, a +12°C heat shock enhanced salinity tolerance (cross-tolerance) but a +15°C heat shock was too extreme and reduced salinity tolerance (cross-susceptibility; Todgham *et al*., 2005). Because responses to individual stressors are often non-linear, these complex multi-stressor patterns are not surprising (Carmichael *et al*., 2025), yet they complicate efforts to incorporate multi-stressor interactions into conservation practice. Thus, to better understand dose-dependent interactions, we recommend studies on cross-tolerance vary the intensity of priming stressors across independent treatments (e.g. Todgham *et al*., 2005; Rodgers and Gomez Isaza, 2022; Jones *et al*., 2024). The temporal aspect of stressor exposure is equally important. The duration, frequency and time between stressful events can influence the development of cross-protection. Short, repeated exposures to a stressor may prime organisms for future challenges, while prolonged or continuous exposure may lead to reduced resilience in some cases. Schunck and Liess (2022) found that the time between sequential stress exposures can turn antagonistic interactions into synergistic ones. In other cases, a recovery period between stress events is required for cross-protection to develop. Tidepool sculpins (*O. maculosus*) required between 8 and 48 h of recovery following heat stress (+12°C) for cross-tolerance to osmotic and hypoxia stress to develop (Todgham *et al*., 2005). Recovery periods may reflect the time required for protective mechanisms, like the mobilization of energy stores, to be expressed. Conversely, insufficient time between stressors can sometimes lead to cumulative stress and reduced resilience (Todgham *et al*., 2005). The sequence in which organisms encounter stressors also matters. Exposure to one stressor may prepare an organism for future stress through shared protective mechanisms. However, the reverse order does not always produce the same effect. For example, when water beetles (*Enochrus jesusarribasi* and *Nebrioporus baeticus*) are exposed to salinity stress, their resistance to desiccation increases, demonstrating cross-protection (Pallarés *et al*., 2017). However, this interaction is not observed when the stress sequence is reversed (Pallarés *et al*., 2017). The order of stress exposure should be carefully considered when designing experiments, interpreting results and managing environmental threats.

In summary, not all forms of stress are equal, and exposure to mild stress is the most likely to produce cross-protection. It cannot be assumed that stressor pairs will always interact in a single way, and research must be done into the modulating factors of stressor intensity, exposure order and the role of recovery periods. Ideally, researchers should aim to mimic stressor dynamics occurring in nature or specific conservation contexts (see 4 below). When designing cross-tolerance experiments, it is important to incorporate a range of stressor intensities, manipulate the order in which stressors are applied, and vary the duration of recovery between exposures. Each of these factors can significantly influence the likelihood and magnitude of protective responses. By understanding these factors, conservation practitioners may be able to develop more effective strategies to enhance species resilience in the face of multiple, emerging stressors.

## Cross-Protection Can be Life Stage-, Sex- and Species-Specific

Research on multiple-stressor scenarios has shown that cross-protection mechanisms are often life stage-dependent (Fitzgerald *et al*., 2016; Fitzgerald *et al*., 2017; Fitzgerald *et al*., 2019; Mutamiswa *et al*., 2019). Priming stressors sometimes only confer protection against subsequent stress during specific life stages, potentially owing to differences in stress sensitivity (Mirón-Gatón *et al*., 2025) and metabolic demands (Komoroske *et al*., 2014; Fitzgerald *et al*., 2017; Del Rio *et al*., 2021). Therefore, it is crucial to study how ontogeny influences cross-protection to avoid incorrect generalizations based on observations from a single life stage. For example, Mirón-Gatón *et al*. (2025) examined the responses of supratidal beetles (*Ochthebius quadricollis* and *Ochthebius lejolisii*) to salinity and desiccation across development stages and found that eggs and larvae were significantly more tolerant of desiccation compared to adults. Exposure to stressors during critical life stages can also induce cross-protective effects on subsequent generations, either through parental influences or transgenerational mechanisms (Plautz *et al*., 2013; Augustyniak *et al*., 2017). When protective effects persist across multiple generations, this phenomenon is termed transgenerational cross-protection (Rodgers and Gomez Isaza, 2023). Understanding these processes is essential for elucidating the evolutionary trajectories of cross-protection, particularly in the context of long-term conservation efforts.

Cross-protection can also be influenced by intraspecific variation among individuals, both among and within a population. For example, sex can play a pivotal role in influencing cross-protection mechanisms (Donaldson *et al*., 2014;Teffer *et al*., 2019; Mottola *et al*., 2022). During the adult stage, mature individuals undergo numerous sex-specific energy-demanding adjustments associated with reproduction and parental care. Thus, it is unsurprising that stress exposure has been shown to enhance phenotypic plasticity in response to a subsequent stressor in a sex-specific manner, with females exhibiting less plasticity than males (Teffer *et al*., 2019; Mottola *et al*., 2022). If cross-protection mechanisms are effective in only one sex, this could have significant demographic and ecological implications. Thus, it is crucial to design sex-balanced experiments to accurately detect sex-specific cross-protection responses.

A stressor eliciting a cross-protective response in one species may not necessarily have the same effect in another (Donaldson *et al*., 2014; Fitzgerald *et al*., 2016; Fitzgerald *et al*., 2017; Mirón-Gatón *et al*., 2025). Ecosystem resistance to stressors often depends on the presence of stress-tolerant species that can compensate for more sensitive species, thereby sustaining essential ecosystem processes, like primary production (Vinebrooke *et al*., 2004). Experimental set-ups, such as microcosms or mesocosms, can offer more comprehensive and realistic scenarios, particularly when conducting research within a conservation framework (see 5 and 10 below).

## Assess the Costs and Trade-Offs of Cross-Protection

Cross-protection effects, while often assumed to be beneficial, can entail fitness trade-offs within and across generations (Padda and Stahlschmidt, 2022). Such fitness trade-offs can stem from the metabolic burden of maintaining broadly protective traits, inherent compromises in trait optimization, or the maladaptive upregulation of stress responses (Morgan *et al*., 1997; Fadhlaoui and Couture, 2016; Loughland and Seebacher, 2020). While few studies have directly measured the costs linked to cross-protection, existing evidence points to notable consequences such as diminished reproductive output, increased mortality, reduced body sizes and lower levels of activity (Plautz *et al*., 2013; Del Rio *et al*., 2019; Scharf *et al*., 2019). For instance, in freshwater snails (*Biomphalaria glabrata*), transgenerational cross-protection has been linked to a decline in offspring production (Plautz *et al*., 2013). Likewise, in the red flour beetle (*Tribolium castaneum*), cold-stress exposure enhances resistance to starvation but simultaneously reduces the likelihood of successful mating (Scharf *et al*., 2019). A recent study by Mottola (2022) also showed that adult zebrafish (*Danio rerio*) exposed to heat stress and copper produced fewer and smaller offspring compared to those reared under a single-stressor scenario or control conditions. The reduction in offspring fitness observed in the zebrafish study may be linked to maternal effects (Mottola, 2022). While the parental generation showed improved or unchanged tolerance to multiple stressors, this plasticity was associated with high embryo mortality and poor egg quality, likely due to an energetic trade-off where resources may have been redirected to enhance protective performance at the expense of reproductive success. Taken together, these findings suggest that cross-protective phenotypes can incur significant energetic costs and may result in fitness trade-offs, which can sometimes appear in subsequent generations. Thus, from a conservation perspective, cross-protection might temporarily decrease the likelihood of population collapse, but in some instances, it may jeopardize the very processes essential for long-term persistence.

We recommend future research prioritizes investigating the potential trade-offs associated with cross-protection by assessing energetic costs (e.g. metabolism and growth rates), performance and behavioural trade-offs (e.g. locomotor performance, predator avoidance behaviour) and fitness and reproductive costs (e.g. offspring size, number and survival) across generations as these trade-offs are sometimes lagged. Understanding these costs may illuminate the selection pressures driving protective interactions, especially regarding how these interactions manifest across generations. The costs of cross-protection might also vary based on the type and severity of the stressor, necessitating continued exploration of cross-protection across diverse stressors and severity scenarios.

## Embrace Temporal Variability in Environmental Stressors

Arguably, the vast majority of ‘stressful’ environmental events occur as ‘pulses’ (typically days to weeks) or ‘acute’ events (minutes to hours). Such events may be repeated over time, either with predictable or more stochastic intervals (Dowd *et al*., 2015), and the magnitude of the fluctuation can also differ from event to event. The frequency and intensity of these events are both predicted to increase through global change (Jentsch *et al*., 2007), with potentially profound consequences for populations. The conservation consequences of such short-term fluctuations are difficult to disentangle from the environmental background, i.e. unless they cause mortality, but it is increasingly clear that they cannot be ignored. Over recent decades there has been a resurgence of interest in the nonlinear effects of temporal environmental fluctuations on physiological function and species performance (reviewed in Cabrerizo and Marañón, 2022, Denny, 2017, Denny, 2019, Ruel and Ayres, 1999). The mathematical predictions of Jensen’s inequality serve as a potential null model for the effects of stressor variation through time, but just as multi-stressor interactions are not uniformly additive, deviations from the purely mathematical predictions are not uncommon (Koussoroplis *et al*., 2017; Denny and Dowd, 2022). For example, stochastic fluctuations—those more reminiscent of patterns in nature—may generate unique results compared to fluctuations of constant amplitude and period, even if both treatments conditions have the same mean and overall variance (Rodgers *et al*., 2018; Nancollas and Todgham, 2022; Brown *et al*., 2024).

Most of this work has focused on single stressors, given the obvious experimental complexities involved in recreating environmentally relevant fluctuations. However, the theory has been extended to two or more stressors by Koussoroplis *et al*. (2017). Empirical studies on two or more fluctuating stressors are still rare, and they most frequently include temperature as one of the factors. Studies on more than two fluctuating, interacting stressors are extremely unusual (Orr *et al*., 2024). Given the potential complexity of multi-stressor interactions outlined in Part 1 above, a critical step in experimental design is to develop a baseline understanding of patterns of stressor variation in the field. This serves at least two practical purposes:

- *Reducing parameter space:* This approach can dramatically reduce the potential parameter space that has to be explored in laboratory studies, saving time and resources and reducing the number of animals needed for experiments. As a simple example, if shifts in salinity and temperature always co-occur in the focal organism’s habitat—rather than being sequential—then only co-occurring stressors need be investigated in the lab.
- *Increasing environmental relevance:* This approach will increase the ecological relevance of laboratory results by more closely approximating natural patterns.

However, achieving these practical benefits can be complicated by patterns of variation in multiple stressors. Embracing temporal variability in stressors is essential for understanding the complex dynamics of stressor interactions. By incorporating realistic temporal patterns into experimental designs, researchers can better predict how organisms will respond to the multifaceted challenges posed by global change. For example, using real-time temperature or hypoxia loggers in the field and using these data to simulate real-world conditions can add a level of realism in laboratory studies (e.g. Fangue *et al*., 2001; Kern *et al*., 2015). This approach not only enhances the ecological validity of studies but also provides more accurate insights for conservation management.

## Biotic Contexts Require More Attention

Cross-protection may be a result of ‘pre-adaptation’ between naturally co-occurring stressors, like elevated temperatures combined with aquatic hypoxia (Rodgers and Gomez Isaza, 2021; Rodgers and Gomez Isaza, 2023). While cross-tolerance has been mostly studied between abiotic stressors, particularly at the individual level, the role of biotic contexts has been largely overlooked (Rodgers and Gomez Isaza, 2023). The few studies that have investigated biotic stressors have found evidence of cross-protection arising from social stress (MacLeod *et al*., 2023), starvation (Lu *et al*., 2019), immune challenges (Sinclair *et al*., 2013), parental influence (Plautz *et al*., 2013) and biotic interactions such as competition and predation (Mueller *et al*., 1993; Janssens and Stoks, 2017; Henry *et al*., 2018; Thompson *et al*., 2018).

Stress can be transmitted (Feugere *et al*., 2023) or buffered (Culbert *et al*., 2019; Gilmour and Bard, 2022) within groups, in turn altering the prevalence of cross-protection mechanisms in social contexts. Additionally, species interaction can mitigate or amplify the combined effects of biotic or abiotic stressors that would otherwise be synergistic (Piscart *et al*., 2007; Brodeur *et al*., 2015; Lange and Marshall, 2017). The type of interaction (e.g. synergistic or antagonistic) may vary across biological organization levels and the species’ position in trophic networks (Beauchesne *et al*., 2021). For instance, cross-tolerance between stressors may only appear at the population, community or ecosystem levels (Jackson *et al*., 2021; Zhou and Wang, 2023). Consequently, overlooking integrative approaches may under- or overestimate the prevalence of cross-protection in natural ecosystems (Galic *et al*., 2018; Orr *et al*., 2020). For example, higher biodiversity may benefit ecosystem resilience through species co-tolerance (Vinebrooke *et al*., 2004). Therefore, we advocate for future research to implement integrative community physiology approaches considering social and biotic contexts (e.g. stressors such as competition, predator cues, pathogens and hierarchical conflicts). This approach will help refine our understanding of the role that cross-protection plays in determining an ecosystem’s resilience to environmental change.

## Cross-Tolerance Can Occur among Emerging Stressors

Ongoing environmental change is seeing a growing list of stressors across habitats worldwide. Most concerning is the growing risk of emerging or anthropogenic stressors that have unknown consequences on biota (Reid *et al*., 2019). Yet, ecological surprises have been observed where cross-protection interactions occur among human-driven stressors like pharmaceuticals, pesticides and microplastics (e.g. Dolci *et al*., 2013; Augustyniak *et al*., 2017; Alzahrani and Ebert, 2018; Schunck and Liess, 2022; Chang *et al*., 2023; Jones *et al*., 2024). Most common is the documented occurrence of cross-tolerance interactions between chemical compounds (pesticides and pharmaceuticals) that share the same mode of action (Augustyniak *et al*., 2017; Schunck and Liess, 2022; Jones *et al*., 2024). Cross-tolerance effects have also been documented between natural and anthropogenic stressors (Rodgers and Gomez Isaza, 2021). Exposure to natural stressors like heat, hypoxia and salinity can enhance resistance to pesticides and pollutants in various species, including crustaceans, fish and amphibians (Dolci *et al*., 2013; Dolci *et al*., 2014; Fitzgerald *et al*., 2016; Dolci *et al*., 2017; Alzahrani and Ebert, 2018; Wang *et al*., 2020; Schunck and Liess, 2022). These examples of cross-tolerance among natural and novel stressors contribute to optimism in an era of rapid and complex environmental change. However, we are far from reaching a predictive understanding of which novel stressors will (and will not) interact to provide cross-tolerance benefits. It is therefore crucial that we understand the impacts that emerging stressors (e.g. micro- and nanoplastics, ocean acidification, poly- and perfluoroalkyl substances [PFAS], noise and light pollution) may impose on animals, what levels cause harm and which stressors show cross-tolerance, to enable predictions of global change impacts and the prioritization of stressor management for conservation.

## The Search for Underlying Mechanisms Is Difficult but Necessary

Although the occurrence of cross-protection interactions has been increasingly reported in multi-stressors studies (Rodgers and Gomez Isaza, 2021), identifying the underlying mechanisms is fraught with complexity. Cross-protection interactions are thought to arise when both stressors activate shared protective mechanisms or signaling pathways (Sinclair *et al*., 2013; Rodgers and Gomez Isaza, 2023) that are involved in the generalized stress response. Most studies have focused on key cellular protective mechanisms involving, for example, heat shock proteins, HIF-1, reactive oxygen species (ROS) (Hermes-Lima *et al*., 2001; Hermes-Lima and Zenteno-Savín, 2002; Perez and Brown, 2014; Giraud-Billoud *et al*., 2019) and metabolite accumulation (Benoit *et al*., 2009). However, the detection of these mechanisms is often more complex than it seems (Gotcha *et al*., 2018; Mottola *et al*., 2022). Mechanisms can vary across temporal scales: for instance, some mechanisms may be upregulated or downregulated depending on the duration of stress exposure. The order and intensity of stressors also play a role, and adjustments may occur across multiple levels of biological organization, from genes to whole-animal levels (Anttila *et al*., 2015; Terry *et al*., 2024).

Studies focusing on only a few traits or pathways may only provide a fragmented comprehension of cross-tolerance mechanisms. We need to move toward global approaches that allow for the simultaneous measurement of multiple traits and potential mechanisms across several levels of biological organization (e.g. Collins *et al*., 2021, Hůla *et al*., 2022, Terry *et al*., 2024; see Section 7). These approaches can better identify how multiple mechanisms interact together to achieve increased stress tolerance. Improving our mechanistic understanding is crucial to elucidate the adaptive potential and trade-offs/costs of cross-protection interaction. This understanding would strongly guide our conservation efforts and help harness the power of cross-protection mechanisms to promote species resilience to current and future global change stressors (Rodgers and Gomez Isaza, 2021).

## Emerging Approaches Can Facilitate Cross-Tolerance Investigations

Emerging approaches, such as (multi)omics, artificial intelligence (AI) and large-scale meta-analyses can complement existing classical laboratory approaches to (i) understand complex physiological mechanisms, (ii) identify environmental drivers of priority concern and (iii) inform management decisions for effective conservation actions in natural ecosystems (Côté *et al*., 2016). While some mechanisms of cross-tolerance have been proposed (e.g. transcriptional frontloading), global high-throughput omics (e.g. functional genomics, proteomics or epigenomics) can help address biodiversity challenges (De León *et al*., 2023) through the discovery of novel underpinning mechanisms (Rey *et al*., 2020; Collins *et al*., 2021; Lemos, 2021; Collins *et al*., 2023). Likewise, greater attempts to integrate such approaches, using multi-level datasets from genome to phenome, are needed to better characterize the functional mechanisms of cross-tolerance, in turn assisting with conservation (Tills *et al*., 2023; Madeira *et al*., 2024).

Investigating cross-tolerance in a conservation context also requires large-scale meta-analyses (e.g. Orr *et al*., 2024) to accurately and quantitatively synthesize non-additive interactions between drivers (Piggott *et al*., 2015). For example, a meta-analysis by Velasco *et al*. (2018) revealed that antagonistic interactions were highly prevalent for particular stressor pairs (salinity + toxicants), habitat types (transitional waters) and groups (vertebrates). Modeling approaches are continuously being refined to better capture the biological effects of multiple stressors (Alter *et al*., 2024) and may improve the detection of cross-tolerance mechanisms. We also anticipate meta-analyses to become increasingly powerful via the incorporation of AI (e.g. Reason *et al*., 2024), open-access repositories and interactive web applications (e.g. from Orr *et al*., 2024) to assist in (i) automating and accelerating data extraction and analysis, (ii) identifying overlooked combinations of stressors that may confer cross-tolerance and (iii) contributing to the conservation of biodiversity and ecosystems (Kwok, 2019). As open science is critical for conservation biology (Roche *et al*., 2022), we urge researchers to utilize the aforementioned emerging approaches and share their data in order to accelerate discoveries and our understanding of cross-protection mechanisms.

## Investigate Cross-Protection in the Field

In the previous sections, we have discussed the complexities of observed patterns of cross-protection, the related natural variation and potential approaches to understanding the underlying mechanisms. However, translation of this understanding into conservation action requires that it be applicable in the field. Only in nature will any conservation interventions that rely on knowledge of cross-tolerance be subjected to the full scrutiny of co-varying abiotic parameters, to the nuances of biotic interactions and to the (likely numerous) other factors that are missing from our laboratory studies. As soon as we remove organisms from the wild, we alter them in ways that may or may not change their physiological responses to multiple stressors. Complementing laboratory studies with field studies, or employing intermediate approaches using microcosms or mesocosms, is essential. These approaches can offer a way to assess cross-protection in more ecologically realistic scenarios. For example, intertidal zones can provide valuable study systems as they experience rapidly fluctuating environmental factors (Collins *et al*., 2023). Researchers could examine organismal tolerance to a range of stressors before and after stressful events such as heatwaves or algal blooms. Methods such as field critical thermal maxima, field metabolic rate, biotelemetry and portable point of care devices will be crucial in successfully investigating cross-protection in natural settings.

In conservation, we often focus on the removal or mitigation of stressors within a habitat, whereas many laboratory-based studies only investigate the addition of stressors. It is crucial to understand how the removal or mitigation of a stressor impacts cross-tolerance. This requires adaptive management—an approach that emphasizes on flexibility and learning-by-doing (Lima *et al*., 2023; Tudor *et al*., 2023). To implement this approach effectively, we recommend researchers first identify the primary stressors affecting the target species and habitats. Establishing baseline physiological and ecological data for the target species under current conditions is also important. Continuous monitoring of the species and habitats after removing or mitigating stressors helps assess changes in cross-tolerance and overall health. Based on the monitoring data, we recommend management strategies be adjusted to optimize conservation outcomes. For example, if removing a pollutant from an intertidal zone, researchers should observe the responses of resident species before and after the intervention, noting any changes in their tolerance to other stressors such as temperature fluctuations or salinity changes. However, this type of management approach is yet to be implemented. By integrating field studies with adaptive management, we can potentially develop more effective conservation strategies that account for the complexities of natural environments. This approach will help ensure that our interventions are not only theoretically sound but also practically effective in promoting species resilience and ecosystem health in the face of multiple stressors.

## Practical Applications of Cross-Protection for Conservation

Cross-protection represents a powerful, yet underutilized tool in conservation biology. Once a greater understanding of cross-protection is realized, these interactions could be strategically targeted and applied, e.g. by:

### Stress priming

One particularly exciting aspect of cross-protection is the potential for stress priming, defined as the process of inducing stress tolerance in organisms by exposing them to sublethal levels of stress (Hilker *et al*., 2016). This approach aims to enhance an organisms’ resilience to future, more severe stress. Stress priming has been extensively applied in coral reef restoration, where exposing corals and their symbionts to heat stress can increase their resilience to subsequent bleaching events (Hackerott *et al*., 2021). Although research on stress priming involving heterologous stressors is still emerging, there is evidence that exposing corals to salinity stress can enhance thermal tolerance (Ochsenkühn *et al*., 2017). Similarly, stress priming has been investigated in an aquaculture context where fish are prepared for stressful events, like transfer to sea pens or heat stress (Pettinau *et al*., 2022; Rodgers and Gomez Isaza, 2022). For example, Dubeau *et al*. (1998) primed juvenile Atlantic salmon (*Salmo salar*) with mild heat stress (26°C for 15 min) 3 h before they were transferred to salt water, finding that heat-primed fish showed significantly improved survival. Stress priming could play a pivotal role in ‘engineering’ stress resilient populations, particularly for imperiled species where restocking programs are already in use. However, it remains unclear how long stress priming effects last and if repeated stress priming is required to maintain protective effects.

### Captive breeding and reintroduction programs

Cross-protection could also be applied in captive breeding programs, where animals are exposed to mild stressors that mimic conditions they will face in the wild. For example, small changes in environmental conditions or mild disease exposure may help animals in captivity develop cross-protection and better prepare them for reintroduction into the wild, increasing their chances of survival post-release. Stress priming may be particularly valuable for species reintroduced or translocated to ecosystems that have changed drastically since their removal, or habitats that are under high levels of environmental stress. The use of cross-protection has been taken up more readily in plants than animals, with many studies investigating how cross-protection can be used to grow drought-tolerant crops (Walter *et al*., 2013; Liu *et al*., 2022; Kashyap *et al*., 2024). Stress priming of seeds is widely used to enhance stress tolerance, with several priming protocols being patented (e.g. EasyPrime, ThermocureTM, IMPROVERTM; Paparella *et al*., 2015). Stress priming in crops is seen as a more socially acceptable and affordable method of enhancing stress tolerance compared to alternatives like genetic engineering and chemical applications (Ganie *et al*., 2024).

### Boosting climate change resilience

Climate change brings multiple concurrent changes, such as heat waves, droughts and altered rainfall patterns, which pose significant threats to many species (Reid *et al*., 2019). However, some species may develop cross-tolerance to cope with these changes (Del Rio *et al*., 2019). Conservationists may be able to leverage cross-tolerance mechanisms by manipulating environments to gradually expose species to multiple stressors, thereby improving their chances of survival in a rapidly changing world. This could potentially include controlled breeding programs or relocation to areas with more predictable future climates. To effectively implement stress priming, it is crucial to identify the correct dose of the initial stressor and ensure that deleterious trade-offs are minimized.

### Genetic conservation and evolutionary contexts

Cross-tolerance can operate across various organizational levels (e.g. individual, population, community; Sections 2, 4 above) and can be extended to an evolutionary context (Zhou and Wang, 2023) through transgenerational cross-protection (Rodgers and Gomez Isaza, 2021). Certain individuals or populations within a species are therefore more likely to better mount a stress response and have adaptations to tolerate a range of environmental conditions. This genetic diversity could be critical for the survival of endangered species, especially when facing a combination of stressors. By prioritizing genetic diversity and selecting for stress-tolerant individuals in conservation programs, cross-protection can be encouraged through artificial selection, increasing the likelihood that populations remain capable of adapting to a variety of challenges (He *et al*., 2016; Caruso *et al*., 2021; Rodgers and Gomez Isaza, 2024). Conservation strategies like seed banks, genetic repositories, selective reintroductions and the protection of wild genetic resources could be crucial for preserving this diversity and fostering cross-tolerant individuals.

## Conclusion

In the Anthropocene, species are increasingly exposed to a multitude of stressors that challenge their survival. Understanding cross-protection mechanisms offers a promising avenue for enhancing species resilience and informing conservation strategies. Here, we have outlined 10 key considerations for investigating cross-protection in a conservation context, emphasizing the importance of stressor intensity, timing, species-specific responses and the integration of emerging approaches like omics and meta-analyses.

We highlight that cross-tolerance interactions are complex and influenced by various factors, including life-stage, sex and temporal variability of stressors. While laboratory studies provide valuable mechanistic insights, field investigations are crucial to fully understand how these interactions play out in natural environments. Moreover, recognizing the potential trade-offs associated with cross-tolerance is essential for developing effective, long-term conservation management plans.

By leveraging cross-tolerance mechanisms, conservation practitioners may be able to develop more robust strategies to mitigate the impacts of emerging stressors and enhance species resilience. This approach not only aids in preserving biodiversity but also provides a framework for adaptive management in the face of rapid environmental change. Future research should continue to explore the underlying mechanisms of cross-protection, quantify potential trade-offs and apply these insights to real-world conservation challenges. In conclusion, cross-protection represents a powerful tool in the conservation toolkit, offering novel solutions to enhance species resilience and ensure the persistence of biodiversity in an increasingly unpredictable world.

## References

[ref1] Alter K , Jacquemont J, Claudet J, Lattuca ME, Barrantes ME, Marras S, Manríquez PH, González CP, Fernández DA, Peck MA et al. (2024) Hidden impacts of ocean warming and acidification on biological responses of marine animals revealed through meta-analysis. Nat Commun 15: 2885–2813. 10.1038/s41467-024-47064-3. 38570485 PMC10991405

[ref2] Alzahrani SM , Ebert PR (2018) Stress pre-conditioning with temperature, UV and gamma radiation induces tolerance against phosphine toxicity. PloS One 13: e0195349. 10.1371/journal.pone.0195349. 29672544 PMC5909616

[ref3] Anttila K , Lewis M, Prokkola JM, Kanerva M, Seppanen E, Kolari I, Nikinmaa M (2015) Warm acclimation and oxygen depletion induce species-specific responses in salmonids. J Exp Biol 218: 1471–1477. 10.1242/jeb.119115. 25827840

[ref4] Augustyniak M , Tarnawska M, Babczyńska A, Kafel A, Zawisza-Raszka A, Adamek B, Płachetka-Bożek A (2017) Cross tolerance in beet armyworm: long-term selection by cadmium broadens tolerance to other stressors. Ecotoxicology (London) 26: 1408–1418. 10.1007/s10646-017-1865-5. 29058177

[ref5] Beauchesne D , Cazelles K, Archambault P, Dee LE, Gravel D, Wootton T (2021) On the sensitivity of food webs to multiple stressors. Ecol Lett 24: 2219–2237. 10.1111/ele.13841. 34288313

[ref113] Benoit JB , Lopez-Martinez G, Elnitsky MA, Lee Jr RE, Denlinger DL (2009) Dehydration-induced cross tolerance of Belgica antartica larvae to cold and heat is facilitated by trehalose accumulation. Physiol A Mol Integr Physiol 152: 518–523. 10.1016/j.cbpa.2008.12.009. 19141330

[ref6] Brodeur MC , Piehler MF, Fodrie FJ (2015) Consumers mitigate heat stress and nutrient enrichment effects on eelgrass *Zostera marina* communities at its southern range limit. Mar Ecol Prog Ser 525: 53–64. 10.3354/meps11186.

[ref7] Brown S , Rivard GR, Gibson G, Currie S (2024) Warming, stochastic diel thermal fluctuations affect physiological performance and gill plasticity in an amphibious mangrove fish. J Exp Biol 227: jeb246726. 10.1242/jeb.246726. 38904077

[ref8] Burleson ML , Silva PE (2011) Cross tolerance to environmental stressors: effects of hypoxic acclimation on cardiovascular responses of channel catfish (*Ictalurus punctatus*) to a thermal challenge. J Therm Biol 36: 250–254. 10.1016/j.jtherbio.2011.03.009. 21666848 PMC3110708

[ref9] Cabrerizo MJ , Marañón E (2022) Net effect of environmental fluctuations in multiple global-change drivers across the tree of life. Proceedings of the National Academy of Sciences - PNAS 119: 1–8. 10.1073/pnas.2205495119. PMC9371701 35914141

[ref10] Carmichael H , Warfield R, Yvon-Durocher G (2025) Reconciling variability in multiple stressor effects using environmental performance curves. Ecol Lett 28: e70065. 10.1111/ele.70065. 39824762 PMC11741915

[ref11] Caruso C , Hughes K, Drury C (2021) Selecting heat-tolerant corals for proactive reef restoration. Front Mar Sci 8: 632027. 10.3389/fmars.2021.632027.

[ref12] Chang M , Li M, Xu W, Li X, Liu J, Stoks R, Zhang C (2023) Microplastics increases the heat tolerance of daphnia magna under global warming via hormetic effects. Ecotoxicol Environ Saf 249: 114416–114416. 10.1016/j.ecoenv.2022.114416. 38321694

[ref13] Collins M , Clark MS, Spicer JI, Truebano M (2021) Transcriptional frontloading contributes to cross-tolerance between stressors. Evolutionary Applications 14: 577–587. 10.1111/eva.13142. 33664796 PMC7896706

[ref14] Collins M , Clark MS, Truebano M (2023) The environmental cellular stress response: the intertidal as a multistressor model. Cell Stress Chaperones 28: 467–475. 10.1007/s12192-023-01348-7. 37129699 PMC10469114

[ref15] Côté IM , Darling ES, Brown CJ (2016) Interactions among ecosystem stressors and their importance in conservation. Proceedings of the Royal Society. B, Biological sciences 283: 20152592–20152599. 10.1098/rspb.2015.2592. PMC4760168 26865306

[ref16] Culbert BM , Gilmour KM, Balshine S (2019) Social buffering of stress in a group-living fish. Proceedings of the Royal Society B, Biological sciences 286: 20191626–20191626. 10.1098/rspb.2019.1626. PMC6742999 31506060

[ref17] De León LF , Silva B, Avilés-Rodríguez KJ, Buitrago-Rosas D (2023) Harnessing the omics revolution to address the global biodiversity crisis. Curr Opin Biotechnol 80: 102901–102901. 10.1016/j.copbio.2023.102901. 36773576

[ref18] Del Rio AM , Davis BE, Fangue NA, Todgham AE (2019) Combined effects of warming and hypoxia on early life stage Chinook salmon physiology and development. *Conservation*. Physiology 7: coy078. 10.1093/conphys/coy078. PMC6387995 30834124

[ref19] Del Rio AM , Mukai GN, Martin BT, Johnson RC, Fangue NA, Israel JA, Todgham AE (2021) Differential sensitivity to warming and hypoxia during development and long-term effects of developmental exposure in early life stage Chinook salmon. *Conservation*. Physiology 9: 1-coab054. 10.1093/conphys/coab054. PMC8271147 34257996

[ref20] Denny M (2017) The fallacy of the average: on the ubiquity, utility and continuing novelty of Jensen's inequality. J Exp Biol 220: 139–146. 10.1242/jeb.140368. 28100801

[ref21] Denny M (2019) Performance in a variable world: using Jensen's inequality to scale up from individuals to populations. *Conservation*. Physiology 7: coz053. 10.1093/conphys/coz053. PMC6736373 31528348

[ref22] Denny MW , Dowd WW (2022) Elevated salinity rapidly confers cross-tolerance to high temperature in a splash-pool copepod. Integrative Organismal Biology 4: obac037. 10.1093/iob/obac037. PMC9394168 36003414

[ref23] Dolci GS , Dias VT, Roversi K, Roversi K, Pase CS, Segat HJ, Teixeira AM, Benvegnú DM, Trevizol F, Barcelos RCS et al. (2013) Moderate hypoxia is able to minimize the manganese-induced toxicity in tissues of silver catfish (*Rhamdia quelen*). Ecotoxicol Environ Saf 91: 103–109. 10.1016/j.ecoenv.2013.01.013. 23433555

[ref24] Dolci GS , Rosa HZ, Vey LT, Pase CS, Barcelos RCS, Dias VT, Loebens L, Dalla Vecchia P, Bizzi CA, Baldisserotto B et al. (2017) Could hypoxia acclimation cause morphological changes and protect against mn-induced oxidative injuries in silver catfish (*Rhamdia quelen*) even after reoxygenation? Environ Pollut 224: 466–475. 10.1016/j.envpol.2017.02.027. 28238574

[ref25] Dolci GS , Vey LT, Schuster AJ, Roversi K, Roversi K, Dias VT, Pase CS, Barcelos RCS, Antoniazzi CTD, Golombieski JI et al. (2014) Hypoxia acclimation protects against oxidative damage and changes in prolactin and somatolactin expression in silver catfish (*Rhamdia quelen*) exposed to manganese. Aquat Toxicol 157: 175–185. 10.1016/j.aquatox.2014.10.015. 25456232

[ref26] Donaldson MR , Hinch SG, Jeffries KM, Patterson DA, Cooke SJ, Farrell AP, Miller KM (2014) Species- and sex-specific responses and recovery of wild, mature pacific salmon to an exhaustive exercise and air exposure stressor. Comp Biochem Physiol A Mol Integr Physiol 173: 7–16. 10.1016/j.cbpa.2014.02.019. 24607368

[ref27] Dowd WW , King FA, Denny MW (2015) Thermal variation, thermal extremes and the physiological performance of individuals. J Exp Biol 218: 1956–1967. 10.1242/jeb.114926. 26085672

[ref28] Dubeau SF , Pan F, Tremblay GC, Bradley TM (1998) Thermal shock of salmon in vivo induces the heat shock protein hsp 70 and confers protection against osmotic shock. Aquaculture 168: 311–323. 10.1016/S0044-8486(98)00358-5.

[ref29] Ely BR , Lovering AT, Horowitz M, Minson CT (2014) Heat acclimation and cross tolerance to hypoxia. Temperature 1(2): 107–114, 10.4161/temp.29800 PMC4977168 27583292

[ref30] Fadhlaoui M , Couture P (2016) Combined effects of temperature and metal exposure on the fatty acid composition of cell membranes, antioxidant enzyme activities and lipid peroxidation in yellow perch (*Perca flavescens*). Aquat Toxicol 180: 45–55. 10.1016/j.aquatox.2016.09.005. 27649097

[ref31] Fangue NA , Flaherty KE, Rummer JL, Cole G, Hansen KS, Hinote R, Noel BL, Wallman H, Bennett WA (2001) Temperature and hypoxia tolerance of selected fishes from a hyperthermal rockpool in the dry tortugas, with notes on diversity and behavior. Caribbean Journal of Science 37: 81–87.

[ref32] Feugere L , Bates A, Emagbetere T, Chapman E, Malcolm LE, Bulmer K, Hardege J, Beltran-Alvarez P, Wollenberg Valero KC (2023) Heat induces multiomic and phenotypic stress propagation in zebrafish embryos. Proceedings of the National Acamdecy of Science 2: pgad137–pgad137. 10.1093/pnasnexus/pgad137 PMC10205475 37228511

[ref33] Fitzgerald JA , Jameson HM, Dewar Fowler VH, Bond GL, Bickley LK, Uren Webster TM, Bury NR, Wilson RJ, Santos EM (2016) Hypoxia suppressed copper toxicity during early development in zebrafish embryos in a process mediated by the activation of the hif signaling pathway. Environ Sci Tech 50: 4502–4512. 10.1021/acs.est.6b01472. 27019216

[ref34] Fitzgerald JA , Katsiadaki I, Santos EM (2017) Contrasting effects of hypoxia on copper toxicity during development in the three-spined stickleback (*Gasterosteus aculeatus*). Environmental Pollution (1987) 222: 433–443. 10.1016/j.envpol.2016.12.008. 28017364

[ref35] Fitzgerald JA , Urbina MG, Rogers NJ, Bury NR, Katsiadaki I, Wilson RW, Santos EM (2019) Sublethal exposure to copper supresses the ability to acclimate to hypoxia in a model fish species. Aquat Toxicol 217: 105325. 10.1016/j.aquatox.2019.105325. 31711009 PMC6891231

[ref36] Galic N , Sullivan LL, Grimm V, Forbes VE, Vasseur D (2018) When things don't add up: quantifying impacts of multiple stressors from individual metabolism to ecosystem processing. Ecol Lett 21: 568–577. 10.1111/ele.12923. 29460496

[ref37] Ganie SA , McMulkin N, Devoto A (2024) The role of priming and memory in rice environmental stress adaptation: current knowledge and perspectives. Plant, Cell and Environment 47: 1895–1915. 10.1111/pce.14855. 38358119

[ref38] Gilmour KM , Bard B (2022) Social buffering of the stress response: insights from fishes. Biol Lett 18: 20220332–20220332. 10.1098/rsbl.2022.0332. 36285460 PMC9597401

[ref39] Giraud-Billoud M , Rivera-Ingraham GA, Moreira DC, Burmester T, Castro-Vazquez A, Carvajalino-Fernández JM, Dafre A, Niu C, Tremblay N, Paital B et al. (2019) Twenty years of the ‘preparation for oxidative stress’ (pos) theory: ecophysiological advantages and molecular strategies. Comparative Biochemistry and Physiology -Part A : Molecular and Integrative Physiology 234: 36–49. 10.1016/j.cbpa.2019.04.004. 30978470

[ref40] Gotcha N , Terblanche JS, Nyamukondiwa C (2018) Plasticity and cross-tolerance to heterogeneous environments: divergent stress responses co-evolved in an african fruit fly. J Evol Biol 31: 98–110. 10.1111/jeb.13201. 29080375

[ref41] Gunderson AR , Armstrong EJ, Stillman JH (2016) Multiple stressors in a changing world: the need for an improved perspective on physiological responses to the dynamic marine environment. Annual Reviews in Marine Science 8: 357–378. 10.1146/annurev-marine-122414-033953. 26359817

[ref42] Gutierrez MF , Andrade VS, Ale A, Monserrat JM, Roa-Fuentes CA, Herrera-Martínez Y, Bacchetta C, Cazenave J, Rossi AS, Nandini S, Sarma SSS, Piscart C, Wiegand C (2025) Responses of freshwater organisms to multiple stressors in a climate change scenario: a review on small-scale experiments. Environmental Science and Pollution Reserach International 32(8):4431–4444, 10.1007/s11356-025-36034-x. 39903437

[ref43] Hackerott S , Martell HA, Eirin-Lopez JM (2021) Coral environmental memory: causes, mechanisms, and consequences for future reefs. Trends in ecology & evolution (Amsterdam) 36: 1011–1023. 10.1016/j.tree.2021.06.014. 34366170

[ref44] He X , Johansson ML, Heath DD (2016) Role of genomics and transcriptomics in selection of reintroduction source populations. Conserv Biol 30: 1010–1018. 10.1111/cobi.12674. 26756292

[ref45] Henry Y , Renault D, Colinet H (2018) Hormesis-like effect of mild larval crowding on thermotolerance in drosophila flies. J Exp Biol 221: jeb169342. 10.1242/jeb.178681. 29191860

[ref46] Hermes-Lima M , Storey JM, Storey KB (2001) Chapter 20 antioxidant defenses and animal adaptation to oxygen availability during environmental stress. Cell and Molecular Response to Stress Vol 2: 263–287. 10.1016/S1568-1254(01)80022-X.

[ref47] Hermes-Lima M , Zenteno-Savín T (2002) Animal response to drastic changes in oxygen availability and physiological oxidative stress. Comparative Biochemistry and Physiology - C Toxicology and Pharmacology 133: 537–556. 10.1016/S1532-0456(02)00080-7. 12458182

[ref48] Hilker M , Schwachtje J, Baier M, Balazadeh S, Bäurle I, Geiselhardt S, Hincha DK, Kunze R, Mueller-Roeber B, Rillig MC et al. (2016) Priming and memory of stress responses in organisms lacking a nervous system. Biol Rev Camb Philos Soc 91: 1118–1133. 10.1111/brv.12215. 26289992

[ref49] Hůla P , Moos M, Des Marteaux L, Šimek P, Koštál V (2022) Insect cross-tolerance to freezing and drought stress: role of metabolic rearrangement. Proceedings of the Royal Society B: Biological Sciences 289: 20220308. 10.1098/rspb.2022.0308. PMC9174702 35673862

[ref50] Jackson MC , Pawar S, Woodward G (2021) The temporal dynamics of multiple stressor effects: from individuals to ecosystems. Trends in Ecology and Evolution 36: 402–410. 10.1016/j.tree.2021.01.005. 33583600

[ref51] Janssens L , Stoks R (2017) Chlorpyrifos-induced oxidative damage is reduced under warming and predation risk: explaining antagonistic interactions with a pesticide. Environ Pollut 226: 79–88. 10.1016/j.envpol.2017.04.012. 28411497

[ref52] Jentsch A , Kreyling J, Beierkuhnlein C (2007) A new generation of climate-change experiments: events, not trends. Fronteirs in Ecology and the Environment 5: 365–374. 10.1890/1540-9295(2007)5[365:ANGOCE]2.0.CO;2.

[ref53] Jones DK , DiGiacopo DG, Mattes BM, Yates E, Hua J, Hoverman JT, Relyea RA (2024) Naïve and induced tolerance of 15 amphibian populations to three commonly applied insecticides. Aquat Toxicol 272: 106945–106945. 10.1016/j.aquatox.2024.106945. 38759526

[ref54] Kashyap S , Agarwala N, Sunkar R (2024) Understanding plant stress memory traits can provide a way for sustainable agriculture. Plant science (Limerick) 340: 111954–111954. 10.1016/j.plantsci.2023.111954. 38092267

[ref55] Kern P , Cramp RL, Franklin CE (2015) Physiological responses of ectotherms to daily temperature variation. J Exp Biol 218: 3068–3076. 10.1242/jeb.123166. 26254318

[ref56] Komoroske LM , Connon RE, Lindberg J, Cheng BS, Castillo G, Hasenbein M, Fangue NA (2014) Ontogeny influences sensitivity to climate change stressors in an endangered fish. *Conservation*. Physiology 2: cou008-cou008. 10.1093/conphys/cou008. PMC4806739 27293629

[ref57] Koussoroplis A-M , Pincebourde S, Wacker A (2017) Understanding and predicting physiological performance of organisms in fluctuating and multifactorial environments. Ecological monographs 87: 178–197. 10.1002/ecm.1247.

[ref58] Kwok R (2019) AI empowers conservation biology. Nature 567: 133–134. 10.1038/d41586-019-00746-1. 30833739

[ref59] Lange R , Marshall D (2017) Ecologically relevant levels of multiple, common marine stressors suggest antagonistic effects. Sci Rep 7: 6281–6289. 10.1038/s41598-017-06373-y. 28740139 PMC5524789

[ref60] Lemos MFL (2021) Biomarker studies in stress biology: from the gene to population, from the organism to the application. Biology (Basel, Switzerland) 10: 1340. 10.3390/biology10121340. PMC8698987 34943255

[ref61] Lima AC , Sayanda D, Wrona FJ (2023) A roadmap for multiple stressors assessment and management in freshwater ecosystems. Environmental impact assessment review 102: 107191. 10.1016/j.eiar.2023.107191.

[ref62] Liu H , Able AJ, Able JA (2022) Priming crops for the future: rewiring stress memory. Trends Plant Sci 27: 699–716. 10.1016/j.tplants.2021.11.015. 34906381

[ref63] Loughland I , Seebacher F (2020) Differences in oxidative status explain variation in thermal acclimation capacity between individual mosquitofish (Gambusia holbrooki). Functional Ecology 34: 1380–1390. 10.1111/1365-2435.13563.

[ref64] Lu DL , Ma Q, Wang J, Li LY, Han SL, Limbu SM, Li DL, Chen LQ, Zhang ML, Du ZY (2019) Fasting enhances cold resistance in fish through stimulating lipid catabolism and autophagy. J Physiol 597: 1585–1603. 10.1113/JP277091. 30615194 PMC6418777

[ref65] MacLeod KJ , English S, Ruuskanen SK, Taborsky B (2023) Stress in the social context: a behavioural and eco-evolutionary perspective. J Exp Biol 226: jeb245829. 10.1242/jeb.245829. PMC10445731 37529973

[ref66] Madeira D , Madeira C, Calosi P, Vermandele F, Carrier-Belleau C, Barria-Araya A, Daigle R, Findlay HS, Poisot T (2024) Multilayer biological networks to upscale marine research to global change-smart management and sustainable resource use. Sci Total Environ 944: 173837. 10.1016/j.scitotenv.2024.173837. 38866145

[ref67] Maloyan A , Eli-Berchoer L, Semenza GL, Gerstenblith G, Stern MD, Horowitz M (2005) Hif-1alpha-targeted pathways are activated by heat acclimation and contribute to acclimation-ischemic cross-tolerance in the heart. Physiol Genomics 23(1): 79–88, 10.1152/physiolgenomics.00279.2004. 16046617

[ref68] Mirón-Gatón JM , Pallarés S, García-Meseguer AJ, Millán A, Velasco J (2025) Effects of salinity on desiccation resistance in supratidal beetles: cross-tolerance or cross-susceptibility? Estuar Coast Shelf Sci 314: 109131. 10.1016/j.ecss.2025.109131.

[ref69] Morgan JD , Sakamoto T, Grau EG, Iwama GK (1997) Physiological and respiratory responses of the Mozambique tilapia (Oreochromis mossambicus) to salinity acclimation. Comparative Biochemistry and Physiology - A Physiology 117: 391–398. 10.1016/S0300-9629(96)00261-7.

[ref70] Mottola G (2022) The Phenotypic Plasticity of Thermal Tolerance and its Modulation in a Complex Environmental Scenario. University of Turku, Turku, Finland

[ref71] Mottola G , Nikinmaa M, Anttila K (2022) Copper exposure improves the upper thermal tolerance in a sex-specific manner, irrespective of fish thermal history. Aquat Toxicol 246: 106145. 10.1016/j.aquatox.2022.106145. 35338914

[ref72] Mueller LD , Graves JL, Rose MR (1993) Interactions between density-dependent and age-specific selection in drosophila melanogaster. Functional Ecology 7: 469–479. 10.2307/2390034.

[ref73] Mutamiswa R , Machekano H, Chidawanyika F, Nyamukondiwa C (2019) Life-stage related responses to combined effects of acclimation temperature and humidity on the thermal tolerance of *Chilo partellus* (swinhoe) (lepidoptera: Crambidae). J Therm Biol 79: 85–94. 10.1016/j.jtherbio.2018.12.002. 30612691

[ref74] Nancollas SJ , Todgham AE (2022) The influence of stochastic temperature fluctuations in shaping the physiological performance of the California mussel, *Mytilus californianus*. J Exp Biol 225: jeb243729. 10.1242/jeb.243729. 35749162

[ref75] Ochsenkühn MA , Röthig T, D'Angelo C, Wiedenmann J, Voolstra CR (2017) The role of floridoside in osmoadaptation of coral-associated algal endosymbionts to high-salinity conditions. Sci Adv 3: e1602047. 10.1126/sciadv.1602047. 28835914 PMC5559212

[ref76] Orr JA , Macaulay SJ, Mordente A, Burgess B, Albini D, Hunn JG, Restrepo-Sulez K, Wilson R, Schechner A, Robertson AM et al. (2024) Studying interactions among anthropogenic stressors in freshwater ecosystems: a systematic review of 2396 multiple-stressor experiments. Ecol Lett 27: e14463-n/a. 10.1111/ele.14463. 38924275

[ref77] Orr JA , Vinebrooke RD, Jackson MC, Kroeker KJ, Kordas RL, Mantyka-Pringle C, van den Brink PJ, de Laender F, Stoks R, Holmstrup M et al. (2020) Towards a unified study of multiple stressors: divisions and common goals across research disciplines. Proceedings of the Royal Society. B, Biological sciences 287: 20200421. 10.1098/rspb.2020.0421. PMC7282922 32370677

[ref78] Padda SS , Stahlschmidt ZR (2022) Evaluating the effects of water and food limitation on the life history of an insect using a multiple-stressor framework. Oecologia 198: 519–530. 10.1007/s00442-022-05115-w. 35067802

[ref79] Pallarés S , Botella-Cruz M, Arribas P, Millán A, Velasco J (2017) Aquatic insects in a multistress environment: cross-tolerance to salinity and desiccation. J Exp Biol 220: 1277–1286. 10.1242/jeb.152108. 28104801

[ref80] Paparella S , Araújo SS, Rossi G, Wijayasinghe M, Carbonera D, Balestrazzi A (2015) Seed priming: state of the art and new perspectives. Plant Cell Rep 34: 1281–1293. 10.1007/s00299-015-1784-y. 25812837

[ref81] Perez LB , Brown PJ (2014) The role of ros signaling in cross-tolerance: from model to crop. Front Plant Sci 5: 1–6. 10.3389/fpls.2014.00754 PMC4274871 25566313

[ref82] Pettinau L , Seppänen E, Sikanen A, Anttila K (2022) Aerobic exercise training with optimal intensity increases cardiac thermal tolerance in juvenile rainbow trout. Front Mar Sci 9: 912720. 10.3389/fmars.2022.912720.

[ref83] Piggott JJ , Townsend CR, Matthaei CD (2015) Reconceptualizing synergism and antagonism among multiple stressors. Ecol Evol 5: 1538–1547. 10.1002/ece3.1465. 25897392 PMC4395182

[ref84] Piscart C , Webb D, Beisel JN (2007) Acanthocephalan parasite increases the salinity tolerance of the freshwater amphipod *Gammarus roeseli* (crustacea: Gammaridae). Naturwissenschaften 94: 741–747. 10.1007/s00114-007-0252-0. 17487466

[ref85] Plautz SC , Guest T, Funkhouser MA, Salice CJ (2013) Transgenerational cross-tolerance to stress: parental exposure to predators increases offspring contaminant tolerance. Ecotoxicol. 22: 854–861. 10.1007/s10646-013-1056-y. 23483328

[ref86] Reason T , Benbow E, Langham J, Gimblett A, Klijn SL, Malcolm B (2024) Artificial intelligence to automate network meta-analyses: four case studies to evaluate the potential application of large language models. PharmacoEconomics 8: 205–220. 10.1007/s41669-024-00476-9. PMC10884375 38340277

[ref87] Reid AJ , Carlson AK, Creed IF, Eliason EJ, Gell PA, Johnson PTJ, Kidd KA, MacCormack TJ, Olden JD, Ormerod SJ et al. (2019) Emerging threats and persistent conservation challenges for freshwater biodiversity. Biol Rev Cambridge Philos Soc 94: 849–873. 10.1111/brv.12480. 30467930

[ref88] Rey O , Eizaguirre C, Angers B, Baltazar-Soares M, Sagonas K, Prunier JG, Blanchet S, Herrel A (2020) Linking epigenetics and biological conservation: towards a conservation epigenetics perspective. Functional Ecology 34: 414–427. 10.1111/1365-2435.13429.

[ref89] Roche DG , O'Dea RE, Kerr KA, Rytwinski T, Schuster R, Nguyen VM, Young N, Bennett JR, Cooke SJ (2022) Closing the knowledge-action gap in conservation with open science. Conserv Biol 36: e13835-n/a. 10.1111/cobi.13835. 34476839 PMC9300006

[ref90] Rodgers EM , Cocherell DE, Nguyen TX, Todgham AE, Fangue NA (2018) Plastic responses to diel thermal variation in juvenile green sturgeon, *Acipenser medirostris*. J Therm Biol 76: 147–155. 10.1016/j.jtherbio.2018.07.015. 30143289

[ref91] Rodgers EM , Gomez Isaza DF (2021) Harnessing the potential of cross-protection stressor interactions for conservation: a review. Conservation Physiology 9: coab037. 35692493 10.1093/conphys/coab037 PMC8193115

[ref92] Rodgers EM , Gomez Isaza DF (2022) Stress history affects heat tolerance in an aquatic ectotherm (Chinook salmon, *Oncorhynchus tshawytscha*). J Therm Biol 106: 103252. 10.1016/j.jtherbio.2022.103252. 35636892

[ref93] Rodgers EM , Gomez Isaza DF (2023) The mechanistic basis and adaptive significance of cross-tolerance: a 'pre-adaptation' to a changing world? J Exp Biol 226: jeb245644. 10.1242/jeb.245644. 37288646

[ref94] Rodgers EM , Gomez Isaza DF (2024) Multiple stressors- physiological responses to multivariate environments. In Alderman SL, Gillis TE eds, Encyclopedia of Fish Physiology (Second edition), Oxford: Academic Press, pp. 185–197, 10.1016/B978-0-323-90801-6.00140-3

[ref95] Ruel JJ , Ayres MP (1999) Jensen’s inequality predicts effects of environmental variation. Trends in Ecology and Evolution 14: 361–366. 10.1016/S0169-5347(99)01664-X. 10441312

[ref96] Scharf I , Wertheimer KO, Xin JL, Gilad T, Goldenberg I, Subach A (2019) Context-dependent effects of cold stress on behavioral, physiological, and life-history traits of the red flour beetle. Insect Science 26: 142–153. 10.1111/1744-7917.12497. 28631879

[ref97] Schulte PM (2014) What is environmental stress? Insights from fish living in a variable environment. J Exp Biol 217: 23–34. 10.1242/jeb.089722. 24353201

[ref98] Schunck F , Liess M (2022) Time between sequential exposures to multiple stress turns antagonism into synergism. Environ Sci Technol 56: 14660–14667. 10.1021/acs.est.2c04345. 36170596

[ref99] Sinclair BJ , Ferguson LV, Salehipour-shirazi G, MacMillan HA (2013) Cross-tolerance and cross-talk in the cold: relating low temperatures to desiccation and immune stress in insects. Integr Comp Biol 53: 545–556. 10.1093/icb/ict004. 23520401

[ref100] Teffer AK , Hinch S, Miller K, Jeffries K, Patterson D, Cooke S, Farrell A, Kaukinen KH, Li S, Juanes F (2019) Cumulative effects of thermal and fisheries stressors reveal sex-specific effects on infection development and early mortality of adult coho salmon (*Oncorhynchus kisutch*). Physiol Biochem Zool 92: 505–529. 10.1086/705125. 31397628

[ref101] Terry CE , Liebzeit JA, Purvis EM, Dowd WW (2024) Interactive effects of temperature and salinity on metabolism and activity of the copepod *Tigriopus californicus*. J Exp Biol 227: jeb248040. 10.1242/jeb.248040. PMC11418200 39155685

[ref102] Thompson PL , MacLennan MM, Vinebrooke RD (2018) Species interactions cause non-additive effects of multiple environmental stressors on communities. Ecosphere (Washington, DC) 9: n/a. 10.1002/ecs2.2518.

[ref103] Tills O , Holmes LA, Quinn E, Everett T, Truebano M, Spicer JI (2023) Phenomics enables measurement of complex responses of developing animals to global environmental drivers. Sci Total Environ 858: 159555. 10.1016/j.scitotenv.2022.159555. 36283519

[ref104] Todgham AE , Schulte PM, Iwama GK (2005) Cross-tolerance in the tidepool sculpin: the role of heat shock proteins. Physiol Biochem Zool 78: 133–144. 10.1086/425205. 15778933

[ref105] Todgham AE , Stillman JH (2013) Physiological responses to shifts in multiple environmental stressors: relevance in a changing world. Integr Comp Biol 53: 539–544. 10.1093/icb/ict086. 23892371

[ref106] Tudor EP , Lewandrowski W, Tomlinson S (2023) Integrating animal physiology into the adaptive management of restored landscapes. Environ Manag 72: 519–528. 10.1007/s00267-023-01800-5. PMC10372129 36781454

[ref107] Velasco J , Gutiérrez-Cánovas C, Botella-Cruz M, Sánchez-Fernández D, Arribas P, Carbonell JA, Millán A, Pallarés S (2018) Effects of salinity changes on aquatic organisms in a multiple stressor context. Philosophical transactions of the Royal Society of London Series B Biological sciences 374: 20180011. 10.1098/rstb.2018.0011. 30509913 PMC6283958

[ref108] Vinebrooke DR , Cottingham KL, Norberg MSJ, Dodson SI, Maberly SC, Sommer U (2004) Impacts of multiple stressors on biodiversity and ecosystem functioning: the role of species co-tolerance. Oikos 104: 451–457. 10.1111/j.0030-1299.2004.13255.x.

[ref109] Walter J , Jentsch A, Beierkuhnlein C, Kreyling J (2013) Ecological stress memory and cross stress tolerance in plants in the face of climate extremes. Environ Exp Bot 94: 3–8. 10.1016/j.envexpbot.2012.02.009.

[ref110] Wang S , You M, Wang C, Zhang Y, Fan C, Yan S (2020) Heat shock pretreatment induced cadmium resistance in the nematode caenorhabditis elegans is depend on transcription factors daf-16 and hsf-1. Environ Pollut 261: 114081. 10.1016/j.envpol.2020.114081. 32062098

[ref111] von Weissenberg E , Mottola G, Uurasmaa TM, Anttila K, Engström-Öst J (2022) Combined effect of salinity and temperature on copepod reproduction and oxidative stress in brackish-water environment. Front Mar Sci 9: 952863. 10.3389/fmars.2022.952863.

[ref112] Zhou L , Wang S (2023) The bright side of ecological stressors. Trends in Ecology and Evolution 38: 568–578. 10.1016/j.tree.2023.01.010. 36906435

